# Fertility of Czech Gay and Straight Men, Women, and Their Relatives: Testing the Sexually Antagonistic Gene Hypothesis

**DOI:** 10.1007/s10508-024-02827-3

**Published:** 2024-03-12

**Authors:** Jakub Fořt, Jaroslav Flegr, Radim Kuba, Šárka Kaňková

**Affiliations:** 1 Department of Zoology, Faculty of Science, Charles University, Viničná 7, 128 00 Prague, Czechia; 2 Department of Philosophy and History of Science, Faculty of Science, Charles University, Prague, Czechia; 3 Department of Biology Education, Faculty of Science, Charles University, Prague, Czechia

**Keywords:** Sexually antagonistic gene hypothesis, Homosexuality, Gay, Lesbian, Fertility, Christians

## Abstract

One proposal for the persistence of homosexuality in the human population is the sexually antagonistic gene hypothesis, which suggests that the lower fertility of homosexual individuals, especially men, may be compensated by higher fertility of their relatives of the opposite sex. To test this hypothesis, we have collected data from 7,312 heterosexual men, 459 gay men, 3,352 heterosexual women, and 79 lesbian women mainly from Czechia. In an online survey, participants answered questions regarding their own as well as their parents’ and grandparents’ fertility. For men, we obtained no significant results except for higher fertility of gay men’s paternal grandmothers, but the magnitude of this effect was very small. For the female sample, we recorded lower fertility of lesbian women’s mothers and fathers. In line with our expectations, both gay men and lesbian women had lower fertility rates than their heterosexual counterparts. Our results are consistent with recent studies which likewise do not support the sexually antagonistic gene hypothesis.

## Introduction

Human homosexuality is by many researchers considered to be an evolutionary paradox. Both male and female homosexuality are heritable, i.e., affected by genes (Ganna et al., 2019), and both gay men and lesbian women have significantly fewer offspring than their straight counterparts (Camperio Ciani et al., 2015; Coome et al., 2020; Fořt et al., 2024; King et al., 2005).^1^ Nevertheless, homosexuality is a relatively common trait in various human populations (Barthes et al., 2015; Crapo, 1995). Over the past decades, researchers formulated several evolutionary hypotheses aimed at explaining the persistence of homosexuality in the human population despite the decreased direct reproduction rates of homosexual individuals, which one would expect to be associated with reduced spread of homosexuality-associated alleles (e.g., Apostolou, 2013; Barthes et al., 2013; Camperio Ciani et al., 2004; Miller, 2000; Wilson, 1975).

One of the proposed explanations developed in recent years is the so-called sexually antagonistic gene hypothesis (SAGH), which is based on the notion of sexually antagonistic selection (Camperio Ciani et al., 2004). Generally speaking, sexually antagonistic selection is a process where genetic variants detrimental to the biological fitness of one sex persist in the population if they confer a reproductive advantage to members of the opposite sex. For the process to operate, this advantage should be significant enough to compensate for the detrimental effects of these genetic variants in the sex that is affected negatively (Gibson et al., 2002). In relation to human homosexuality, this hypothesis states that reduced reproduction rates of homosexual men may be compensated by elevated fertility of their maternal female relatives (i.e., mothers, maternal aunts, and maternal grandmothers).

The increased female fertility anticipated by the SAGH was restricted to maternal relatives because certain evidence suggests that alleles associated with male homosexuality may be located on the X-chromosome (Hamer et al., 1993; Sanders et al., 2015; but see Ganna et al., 2019). Given this reasoning, one might conjecture that beyond the higher fertility of maternal female kin of gay men (as opposed to straight men), we should also see higher fertility in gay men’s maternal aunts and grandmothers than in gay men’s paternal aunts and grandmothers. This is because, usually, a male inherits a copy of his X-chromosome only from his mother, not father, and therefore, only maternal aunts and grandmothers can have the antagonistic genetic variants potentially promoting their fertility. In fact, Camperio Ciani and Pellizzari (2012) have reported a higher fertility of maternal (as opposed to paternal) aunts of gay men.

Nevertheless, research testing the X-linked version of the SAGH yielded conflicting results. While some studies reported increased fertility in gay men’s (as opposed to straight men’s) maternal female relatives (and not paternal or male relatives; Camperio Ciani et al., 2004, 2009; Iemmola & Camperio Ciani, 2009; Rahman et al., 2008 for white men; Semenyna et al., 2017), other researchers found increased fertility in both paternal and maternal female relatives (Gómez Jiménez et al., 2020 for transgender androphilic males) or only in paternal female relatives (King et al., 2005). Moreover, Gómez Jiménez et al. recorded higher fertility also in maternal uncles of transgender androphilic males. This indicates that balancing mechanisms other than the sexually antagonistic selection may be involved. In this context, Miller (2000) proposed the overdominance model, which suggests that the higher fertility of male (regardless of whether paternal or maternal) relatives of gay men could be explained by some sort of heterozygous advantage.

Other studies, however, found no difference in the fertility of maternal female relatives of straight and gay men (Fořt et al., 2024; Gómez Jiménez et al., 2020 for cisgender androphilic males), and some even found decreased fertility of gay men’s mothers (Ablaza et al., 2022; Rahman et al., 2008 for non-white men). Most importantly, a recent meta-analysis found no association between a mother’s fertility and her son’s sexual orientation (Blanchard et al., 2020).

The observation that gay men generally have more older brothers than straight men do (Ablaza et al., 2022; Blanchard & Skorska, 2022) could have an impact on assessments of fertility of gay men’s mothers: It could be artificially increased due to the very existence of these older brothers (Ablaza et al., 2022; Khovanova, 2020). In particular, it is possible that the association between family size and male homosexuality is due to the greater likelihood of finding gay men in larger families rather than a consequence of sexually antagonistic genes leading to homosexuality in one sex and greater fertility in the opposite sex. To address this potential bias, one possible approach would be to examine the fertility of mothers with only firstborn gay and straight sons, because these individuals have no older siblings. The results of studies that used this method are, however, mixed: One study reported, as expected, a higher fertility of gay men’s mothers (Iemmola & Camperio Ciani, 2009) but other studies did not (e.g., Camperio Ciani et al., 2004, 2009). Importantly, two recent studies by Raymond et al. (2023) and Semenyna et al. (2023) suggest that there is no difference in the fertility of mothers of gay and straight men once the birth order is taken into account. This indicates that the higher fertility observed in gay men’s mothers may be the result of them having more sons. For a thorough discussion of older brothers’ influence on their younger brothers’ sexual orientation and a possible immunological mechanism of this influence, see, e.g., Ablaza et al. (2022), Blanchard and Klassen (1997), or Bogaert et al. (2018).

The logic of the SAGH can be in principle applied also to female homosexuality (Luoto et al., 2019). Camperio Ciani et al. (2018) found that the pedigree size and kin fertility of lesbian women were significantly higher than in straight women. If sexually antagonistic selection were responsible for maintaining female homosexuality-associated alleles, we should see increased fertility in lesbian women’s *male* relatives. If some other balancing mechanism were involved (e.g., overdominance; Miller, 2000; Zietsch et al., 2008), we should see increased fertility of either female or both male and female relatives.

Zietsch et al. (2021) published interesting results that offer another insight into possible roles of sexually antagonistic genes (or other balancing factors) in human homosexuality. For heterosexual women, they found no genetic correlation (*r*_g_ = 0.01) between the presence of alleles linked to male same-sex sexual behavior and the number of those women’s children. For those women, they did, however, find a genetic correlation between the presence of alleles linked to male same-sex sexual behavior and the number of their opposite-sex sexual partners (*r*_g_ = 0.32), and even a strong correlation between alleles linked to female same-sex sexual behavior and the number of opposite-sex sexual partners (*r*_g_ = 0.73). For heterosexual men, they found a positive genetic correlation between the number of their children and the presence of alleles linked to both male (*r*_g_ = 0.18) and female (*r*_g_ = 0.30) same-sex sexual behavior (Zietsch et al., 2021, Supplementary material). This genetic evidence supports both the SAGH and the overdominance model (Miller, 2000), i.e., a possession of alleles predisposing individuals of either sex to same-sex sexual behavior seems to be associated with fitness benefits for heterosexual individuals, of both sexes.

Based on these ambiguous findings regarding the fertility of relatives of gay men and lesbian women, we decided to test the SAGH using a large online collected dataset. Our hypotheses were preregistered (https://osf.io/a2rbe) and state that:

1. H1: Mothers of homosexual men will exhibit a higher fertility than mothers of heterosexual men.
2. H2: Mothers of firstborn homosexual men will exhibit a higher fertility than mothers of firstborn heterosexual men.
3. H3: Maternal grandmothers of homosexual men will exhibit a higher fertility than maternal grandmothers of heterosexual men.
4. H4: Maternal grandmothers of homosexual men will exhibit a higher fertility than paternal grandmothers of homosexual men.
5. H5: Homosexual men will exhibit lower fertility (have fewer children) than heterosexual men.

In our preregistration, we stated no specific predictions for women or their relatives, planning to perform the same investigations in the female sample primarily for exploratory purposes. However, it is important to note that if sexual antagonism was a mechanism responsible, or partly responsible, for maintaining female homosexuality in the population, we would expect to observe increased fertility in the fathers of lesbian women, as compared to the fathers of straight women.

## Method

### Participants

Between January 19, 2015, and March 28, 2020, a total of 53,538 participants started the survey session. Of these, we excluded those who were less than 30 years old (*N* = 24,578). From the remainder, some participants did not answer one or both items regarding sexual attraction (*N* = 15,203), while others were excluded as their sexual orientation was classified as other than homosexual or heterosexual (*N* = 2,271). From the remaining number, we have also filtered out one participant who took less than 10 min to fill the questionnaire (as an apparently nonserious responder) as well as participants who reported that their maternal or paternal grandparents had more than nine children (considering them outliers; *N* = 85). From the remainder, we have also excluded those who self-identified as asexual (*N* = 130). Afterward, we have also excluded transgender persons (*N* = 66). From the remainder, we have excluded participants (*N* = 2) who met more than two of the following criteria: being over 80 years old, having more than eight children, reporting more than nine mental disorders, reporting having Alzheimer’s or Parkinson’s disease, and being below the 1st or above the 99th percentile (whole dataset taken as a comparison) in height, weight, or body mass index.

The final sample included 7,312 straight men (*M* = 42.67 years, *SD* = 10.48), 459 gay men (*M* = 40.36 years, *SD* = 8.70), 3,352 straight women (*M* = 41.94 years, *SD* = 9.67), and 79 lesbian women (*M* = 38.68 years, *SD* = 8.27). For definitions of homosexuality and heterosexuality, see Measures. The criterion of including in the sample only participants aged 30 or older was chosen to increase the likelihood that their relatives had already completed their reproductive period. A similar study (Camperio Ciani & Pellizzari, 2012) had for the same reason set as a criterion that the analyzed relatives must be 50 years of age or older (we did not collect data on the relatives’ age). Descriptive statistics on the fertility of gay/lesbian and straight participants and their relatives are summarized in Table 1.

**Table 1:** Descriptive statistics of the fertility of participants and their relatives

			Fertility				
			Participant	Mother	Father	Maternal grandmother	Paternal grandmother
Men	Straight	*N*	6741	7291	7291	6694	6674
		*M*	1.44	2.15	2.13	2.80	2.80
		*SD*	1.16	0.81	0.85	1.47	1.49
	Gay	*N*	407	459	459	426	420
		*M*	0.23	2.20	2.16	2.74	2.95
		*SD*	0.64	0.96	0.88	1.48	1.51
Firstborn men	Straight	*N*	3975	4336	4336	3923	3899
		*M*	1.41	1.95	1.93	2.75	2.72
		*SD*	1.14	0.75	0.78	1.44	1.41
	Gay	*N*	215	238	238	222	218
		*M*	0.28	1.89	1.90	2.66	2.93
		*SD*	0.70	0.76	0.74	1.39	1.44
Women	Straight	*N*	2786	3345	3345	3198	3162
		*M*	1.55	2.20	2.16	2.80	2.82
		*SD*	1.00	0.83	0.86	1.46	1.57
	Lesbian	*N*	47	79	79	74	73
		*M*	1.21	2.09	1.97	2.86	2.84
		*SD*	1.06	0.94	0.91	1.65	1.63
Christian men	Straight	*N*	912	963	963	894	890
		*M*	1.80	2.38	2.38	3.11	3.10
		*SD*	1.28	1.04	1.04	1.65	1.66
	Gay	*N*	50	55	55	51	50
		*M*	0.28	2.16	2.27	2.51	3.30
		*SD*	0.76	0.88	0.93	1.42	1.72
Atheist men	Straight	*N*	4173	4518	4518	4131	4129
		*M*	1.36	2.11	2.09	2.74	2.72
		*SD*	1.10	0.74	0.79	1.42	1.43
	Gay	*N*	240	270	270	250	245
		*M*	0.27	2.17	2.19	2.81	2.84
		*SD*	0.66	0.88	0.89	1.46	1.38

Note: *N* stands for the number of participants who provided data regarding the category of relevance (themselves or the relevant kin category); *M* denotes the mean number of children

To ascertain asexuality, we asked participants this question: “Do you consider yourself an asexual person (you are uninterested in any form of sex)?” (range 1–6, with 1 being “certainly not” and 6 being “certainly yes”). Individuals who answered 5 or 6 were labeled as asexual. During the study (after 25,659 individual responses were collected), we have added another item to assess whether there are any transgender people in our sample, namely the question “Do you sometimes feel that you live in the body of a person of the opposite sex (that you are a transsexual)?” (range 0–100, with 0 being “certainly not” and 100 “certainly yes”). A participant was labeled transgender if their score on this item was 50 or more.

### Procedure

The Qualtrics survey was initially advertised in various traditional and online media by members of the research team (television and radio interviews, etc.). Additional participants were recruited for “a study examining various evolutionary, psychological, and parasitological hypotheses, with focus on sexual life,” through the Facebook page “Lab Bunnies” (www.facebook.com/pokusnikralici). This page is geared toward Czech and Slovak nationals interested in contributing to large citizen science projects, especially studies focused on various aspects of evolutionary psychology. The participant pool was expanded and diversified using a Facebook-based snowball method (Kaňková et al., 2015), where participants were prompted to invite others to join by clicking the “Like” button at both the beginning and the end of the survey. On the first page of the survey, participants were informed about the procedure as follows: “The questionnaire is anonymous and obtained data will be used exclusively for scientific purposes. Your cooperation in the project is voluntary and you can terminate it at any time by closing this web page. You can also skip any uncomfortable questions but most valuable are complete data from each participant.” The survey included a total of 701 questions and the average time needed to complete it was about 89 min (the most common completion time was 72 min). It is important to note that we initially collected the data primarily for a different project. Subsequently, we registered the current project and its hypotheses in the Open Science Framework, followed by the data analysis.

### Measures

*Sociodemographic information:* Participants were asked to indicate their sex, age, height, weight, the number and nature of health disorders, size of childhood place of residence (assessed by “What is the population of the town where you spent most of your childhood?,” response range 1–6 with 1 standing for “less than 1000 inhabitants,” 2 for “1,000 to 5,000 inhabitants,” 3 for “from 5,000 to 50,000 inhabitants,” 4 for “from 50,000 to 100,000 inhabitants,” 5 for “from 100,000 to 500,000 inhabitants,” and 6 for “over 500,000 inhabitants”), the strength of religious belief (assessed as an answer to “Faith in God is greatly important to me,” with answer range 0–100, where 0 stood for “certainly not” and 100 “certainly yes”), and a question regarding religious affiliation (“Your religious belief is”: with options (1) I do not believe in God, (2) I believe in God but I am not a member of any church, (3) Roman Catholic, (4) Evangelical Church of the Czech Brethren, (5) Czechoslovak Hussite Church, (6) other). The option “other” was later (after 49,670 individual responses were collected) split in three separate options: (6) other, (7) Judaism, and (8) Buddhism. Participants who self-identified as belonging to categories (3), (4), or (5) were considered Christians. The number of participants in category “other” (including Judaism and Buddhism) was low (*N* = 2,427).

*Sexual orientation:* Participants were asked two 6-point items: “Are you sexually attracted to same-sex individuals?” and “Are you sexually attracted to other-sex individuals?” (with 1 standing for “certainly not” and 6 for “certainly yes”). Participants who stated their attraction toward same-sex individuals was 1 or 2 and their attraction toward opposite-sex individuals was 5 or 6 were labeled as heterosexual. Vice versa, those who stated their attraction toward same-sex individuals was 5 or 6 and their attraction toward opposite-sex individuals was 1 or 2 were labeled as homosexual. For exploratory analyses, we treated sexual orientation also as an ordinal degree (range 1–6) of attraction toward same-sex individuals.

*Fertility of the participants and their family members:* Apart from the number of their own sons and daughters, participants were asked to indicate the number of their siblings, including their sex, year of birth, and whether they are full siblings, maternal half-siblings, paternal half-siblings, or stepsiblings. Mother’s fertility was then calculated as the sum of full siblings and maternal half-siblings, including those whose sex or year of birth was not stated, plus the participant. Father’s fertility was calculated analogously. The participant’s fertility was calculated as a sum of the participant’s biological daughters and sons. Cases where a participant stated neither the number of daughters nor the number of sons were considered missing values but if the participant indicated only the number of daughters/sons, the number of the participant’s sons/daughters was considered to be zero. Maternal grandmother’s fertility was calculated as the sum of the mother’s older and younger brothers and sisters (plus the mother herself). Cases where the participant did not provide any data on these four categories of mother’s siblings were considered missing values. If the participant provided data on at least one of these four categories, the rest (for which the participant provided no data) were considered to be zero. Fertility of the paternal grandmother was calculated analogously. We considered firstborn all participants who reported no older full or maternal half-siblings (excluding those whose birth order could not be estimated, i.e., siblings with the same year of birth as the participant).

The Qualtrics questionnaire also included a set of psychological, health-related, and sexuality-related measures and separate items (most of which are not relevant to the current paper).

### Data Preparation and Analyses

Data were prepared in Excel and analyzed in Jamovi (The Jamovi Project, 2022) and R (using RStudio; Posit Team, 2023). In analyses that worked with binary sexual orientation, missing data for the covariate “strength of religious belief” were substituted with arithmetic means separately for gay men, lesbian women, straight men, and straight women. In analyses that used the ordinal degree of homosexual attraction, observations with missing data for covariates “strength of religious belief” and “degree of heterosexual attraction” were omitted.

Associations between the participant’s sexual orientation and fertility of relatives (target variables: sexual orientation, mother’s fertility, father’s fertility, paternal grandmother’s fertility, maternal grandmother’s fertility, and participant’s fertility) were tested using partial Kendall’s τ correlation, controlling for age, size of childhood place of residence, and strength of religious belief. This test measures the strength and significance of associations between binary, ordinal, and continuous data irrespective of their distribution shapes and offers the advantage of dispensing with the need for variable transformation: It can be applied uniformly across diverse data types. Specifically, Kendall’s τ quantifies the likelihood of the value of a dependent variable for subject A exceeding that for subject B in case the value of an independent variable for subject A is greater than for subject B. This method not only allows for precise control over confounding variables but also addresses the challenge of varying subject numbers across subsets. Differences in the fertility of paternal versus maternal relatives were assessed using the Wilcoxon signed-rank test. For analyses that worked with the ordinal measure of sexual orientation (degree of homosexual attraction), we again employed partial Kendall’s correlation, controlling for age, size of the childhood place of residence, strength of religious belief, and the degree of heterosexual attraction.

Higher religiosity is generally linked to higher fertility rates (Peri-Rotem, 2016), and it has been suggested that “a decision to produce fewer children could (…) prevent the female relatives of androphilic males from exhibiting the elevated reproduction that the SAGH predicts” (Gómez Jiménez et al., 2020, p. 583). Czech and Slovak populations, similar to other Euro-American populations, typically have low fertility rates. As a part of exploration and to circumvent the possible limitation of a low fertility rate in our sample, we have therefore decided to analyze the fertility of relatives of Christian male participants separately, expecting to see higher fertility. We have taken advantage of the fact that Czechia is one of the most atheist countries (Zuckerman, 2006) and conducted the main analyses separately also for atheist men.

For confirmatory testing of hypotheses concerning the male sample (Hypotheses 1–5 stated in the Introduction), we used one-tailed tests. If the result of a one-tailed test pointed in a direction opposite to what we had expected, we performed the one-tailed test in the originally hypothesized direction. (The relevant *p*s were thus over 0.5.) *p* values (alpha = 0.05) were corrected for multiple hypotheses (together for hypotheses 1, 3, 4) by applying the Benjamini–Hochberg (Benjamini & Hochberg, 1995) correction with false discovery rate set to 0.2 which falls within the recommended range of 0.1–0.25 (McDonald, 2014). We preferred this type of correction over others, such as the Bonferroni’s method, which would lead to an unacceptably high probability of Type II error (for a discussion of this topic, see Nakagawa, 2004). For exploratory analyses, we performed two-tailed tests with standard alpha (0.05) and no correction for multiple tests.

### Departures from Preregistration

The main departure from preregistration concerns the choice of item used to calculate the binary variable of sexual orientation. For this purpose, we originally intended to use the following item: “Do you consider your sexual orientation to be rather homosexual than heterosexual?” (range 1–7, with 1 being “certainly not” and 7 “certainly yes”). We would consider those participants providing answers 1 or 2 as heterosexual and those with answers 6 or 7 as homosexual. Nonetheless, this turned out to be rather suboptimal. There was some proportion of participants who, albeit considering their sexual orientation homosexual/heterosexual (i.e., choosing one or the other extreme of the continuum), indicated sexual attractions that clearly contradicted their self-identified sexual orientation. For example, a participant who considered their sexual orientation homosexual also indicated a minimal possible attraction toward same-sex individuals and a maximal possible attraction toward opposite-sex individuals. We believe this could be due to an insufficient comprehensibility of the item we originally intended to use. Some participants might have simply mistaken one pole of the scale for the other. To eliminate this potential issue, we instead decided to use the two items regarding sexual attraction to determine the binary variable of sexual orientation (see above)—unlike the original item, both these items were formulated in a plain and clear manner, thereby precluding possible confusion.

In the confirmatory part, we did not state how exactly the missing values for the variable “strength of religious belief” are to be substituted. In the study, we substituted them separately for men and women according to their sexual orientation. Due to a low number of lesbian women, we refrained from performing specific analyses separately for Christian and atheist female subsamples and performed those only for men. For the sake of brevity and clarity, we chose not to conduct certain preregistered analyses in the exploratory part of the study. These include, for instance, analyses of the fertility of trans- and bisexual participants, or the sex ratio of firstborn children of mothers of homosexual men.

## Results

### Comparisons of Fertility of Gay/Lesbian and Straight Participants and Their Relatives

In line with our prediction, we found a significant negative association between male participants’ fertility and their sexual orientation–gay men showed lower fertility than straight men (H5: *M*_gay_ = 0.23, *SD*_gay_ = 0.64, *M*_straight_ = 1.44, *SD*_straight_ = 1.16, *τ* = −0.23, *p*_(one-tailed)_ < 0.001). Contrary to predictions of the SAGH (and the corresponding preregistered hypothesis), we found no significant positive association between either a mother’s fertility and her son’s sexual orientation (H1: *M*_gay_ = 2.20, *SD*_gay_ = 0.96, *M*_straight_ = 2.15, *SD*_straight_ = 0.81, *τ* = 0.00, corrected *p*_(one-tailed)_ = 0.966) or between maternal grandmother’s fertility and the participant’s sexual orientation (H3: *M*_gay_ = 2.74, *SD*_gay_ = 1.48, *M*_straight_ = 2.80, *SD*_straight_ = 1.47, *τ* = −0.01, corrected *p*_(one-tailed)_ = 0.966). On the other hand, we found a positive association between the participant’s sexual orientation and paternal grandmother’s fertility (*M*_gay_ = 2.95, *SD*_gay_ = 1.51, *M*_straight_ = 2.80, *SD*_straight_ = 1.49, *τ* = 0.03, *p* < 0.001), i.e., gay men’s grandmothers had higher fertility than straight men’s grandmothers. No other significant associations were observed. For more details, see Table 2.

**Table 2:** Correlation between homosexuality and fertility of participants and their relatives

		Fertility				
		Participant	Mother	Father	Maternal grandmother	Paternal grandmother
Men	Partial Kendall *τ*^a^	−.23	.00	.00	−.01	.03
	*p*	** < .001**^**b**^	.966^b, c^	.570	.966^b, c^	** < .001**
	*N*	7,148	7,750	7,750	7,120	7,094
Firstborn men	Partial Kendall *τ*^a^	−.21	−.02	−.01	−.01	.04
	*p*	** < .001**	.963^b^	.401	.433	** < .001**
	*N*	4,190	4,574	4,574	4,145	4,117
Women	Partial Kendall *τ*^a^	−.03	−.03	−.04	.00	.00
	*p*	**.012**	**.016**	** < .001**	.795	.883
	*N*	2,833	3,424	3,424	3,272	3,235
Christian men	Partial Kendall *τ*^a^	−.26	−.03	.00	−.08	.04
	*p*	** < .001**	.154	.828	** < .001**	.102
	*N*	962	1,018	1,018	945	940
Atheist men	Partial Kendall *τ*^a^	−.21	.00	.02	.02	.03
	*p*	** < .001**	.633	.085	.090	**.006**
	*N*	4,413	4,788	4,788	4,381	4,374

Note: All analyses were controlled for participant’s age, size of childhood place of residence, and strength of religious belief

^a^ Heterosexual participants were assigned 0, and homosexual participants were assigned 1. A positive correlation thus indicates a higher fertility of homosexual participants or their relatives

^b^ These relationships were preregistered for one-tailed test

^c^ *p* values adjusted via Benjamini–Hochberg correction (see Data Preparation and Analyses in the Method section). The original one-tailed *p* values (before correction) are .344 for men’s mothers’ fertility and .785 for men’s maternal grandmothers’ fertility

Contrary to predictions of the SAGH, we observed no significant association between mother’s fertility and her son’s sexual orientation in the subsample of firstborn men (H2: *M*_gay_ = 1.89, *SD*_gay_ = 0.76, *M*_straight_ = 1.95, *SD*_straight_ = 0.75, *τ* = −0.02, *p*_(one-tailed)_ = 0.963). In this subsample, we found two associations that were also observed in the full male sample: a negative association between the fertility of firstborn men and their sexual orientation, with gay men once again exhibiting lower fertility compared to straight men (*M*_gay_ = 0.28, *SD*_gay_ = 0.70, *M*_straight_ = 1.41, *SD*_straight_ = 1.14, *τ* = −0.21, *p* < 0.001). Firstborn gay men’s paternal grandmothers had higher fertility than firstborn straight men’s paternal grandmothers (*M*_gay_ = 2.93, *SD*_gay_ = 1.44, *M*_straight_ = 2.72, *SD*_straight_ = 1.41, *τ* = 0.04, *p* < 0.001), see Table 2.

For women, we found a negative association between their fertility and sexual orientation, lesbian women showing lower fertility than straight women (*M*_lesbian_ = 1.21, *SD*_lesbian_ = 1.06, *M*_straight_ = 1.55, *SD*_straight_ = 1.00, *τ* = −0.03, *p* = 0.012). Moreover, we observed two other negative associations concerning lower fertility of lesbian women’s mothers (*M*_lesbian_ = 2.09, *SD*_lesbian_ = 0.94, *M*_straight_ = 2.20, *SD*_straight_ = 0.83, *τ* = −0.03, *p* = 0.016), and lower fertility of lesbian women’s fathers (*M*_lesbian_ = 1.97, *SD*_lesbian_ = 0.91, *M*_straight_ = 2.16, *SD*_straight_ = 0.86, *τ* = −0.04, *p* < 0.001), as compared to parents of straight women, see Table 2.

### Comparisons of Fertility of Gay and Straight Participants and Their Relatives Separately for Christians and Atheists

We observed no significant differences in the proportions of homosexual and heterosexual men between atheists (*N* = 270 [5.6%] homosexuals, *N* = 4,527 [94.4%] heterosexuals) and Christians (*N* = 55 [5.4%] homosexuals, *N* = 965 [94.6%] heterosexuals; χ^2^ = 0.08, *p* = 0.765). Descriptive statistics regarding fertility in the atheist and Christian subsample are presented in Table 1.

In Christian men, we only observed a significant negative association between their maternal grandmother’s fertility and the participant’s sexual orientation, gay men’s maternal grandmothers having fewer children than straight men’s maternal grandmothers (*M*_gay_ = 2.51, *SD*_gay_ = 1.42, *M*_straight_ = 3.11, *SD*_straight_ = 1.65, *τ* = −0.08, *p* < 0.001), and between the participants’ fertility and their sexual orientation, gay men having lower fertility than straight men (*M*_gay_ = 0.28, *SD*_gay_ = 0.76, *M*_straight_ = 1.80, *SD*_straight_ = 1.28, *τ* = −0.26, *p* < 0.001).

For atheist men, we found a positive association between paternal grandmother’s fertility and the participant’s sexual orientation (*M*_gay_ = 2.84, *SD*_gay_ = 1.38, *M*_straight_ = 2.72, *SD*_straight_ = 1.43, *τ* = 0.03, *p* = 0.006), gay men’s paternal grandmothers showing higher fertility than those of straight men. We also observed a negative association between the participants’ fertility and their sexual orientation, gay men producing fewer children than straight men (*M*_gay_ = 0.27, *SD*_gay_ = 0.66, *M*_straight_ = 1.36, *SD*_straight_ = 1.10, *τ* = −0.21, *p* < 0.001). No other significant association was detected in this subsample. For further details, see Table 2.

### Pairwise Comparisons of Fertility of Maternal and Paternal Kin

Results of Wilcoxon signed-rank paired t tests of fertility differences in the paternal and maternal kin in the male sample are summarized in Table 3. Most importantly, prediction of the X-linked version of the SAGH according to which the maternal grandmothers (*M* = 2.71, *SD* = 1.47) of gay men should be more fertile than the paternal grandmothers (*M* = 2.95, *SD* = 1.53) of gay men was not confirmed (H4: *W* = 17,220.5, *p*_(one-tailed)_ = 0.996). This effect, which contradicts the SAGH, was significant for the subsample of Christian gay men, where paternal grandmothers (*M* = 3.30, *SD* = 1.72) were more fertile than their maternal grandmothers (*M* = 2.52, *SD* = 1.43; *W* = 205, *p* = 0.015). In these pairwise comparisons, there were two other significant relationships: We found the mothers (*M* = 2.15, *SD* = 0.81) of straight men to be more fertile than the fathers (*M* = 2.13, *SD* = 0.85) of straight men (*W* = 214,652.5, *p* = 0.034). This also held for the subsample of atheist straight men whose mothers (*M* = 2.11, *SD* = 0.74) were more fertile than their fathers (*M* = 2.09, *SD* = 0.79; *W* = 92,169, *p* = 0.033). There were no other significant associations.

**Table 3:** Comparisons of fertility of the paternal and maternal kin of participants with paired *t*-tests

	*N*	*M*	*SD*	Wilcoxon *W*	*df*	*p*	Rank biserial correlation	Cohen’s *d*
Gay men’s mother	459	2.20	0.96	1,810.5	458	.604	.06	.05
Gay men’s father		2.16	0.88					
Straight men’s mother	7,291	2.15	0.81	214,652.5	7,290	.**034**	.08	.02
Straight men’s father		2.13	0.85					
Christian gay men’s mother	55	2.16	0.88	0	54	.095	−1.00	.26
Christian gay men’s father		2.27	0.93					
Christian straight men’s mother	963	2.38	1.04	1,470	962	.802	.03	.01
Christian straight men’s father		2.38	1.04					
Atheist gay men’s mother	270	2.17	0.88	562	269	.453	−.12	.02
Atheist gay men’s father		2.19	0.89					
Atheist straight men’s mother	4,518	2.11	0.74	92,169	4,517	.**033**	.10	.03
Atheist straight men’s father		2.09	0.79					
Gay men’s maternal grandmother	412	2.71	1.47	17,220.5	411	.996^a^	−0.18	.12
Gay men’s paternal grandmother		2.95	1.53					
Straight men’s maternal grandmother	6,494	2.79	1.46	5,236,305	6,493	.717	−.01	.00
Straight men’s paternal grandmother		2.80	1.49					
Christian gay men’s maternal grandmother	50	2.52	1.43	205	49	.**015**	−.45	.37
Christian gay men’s paternal grandmother		3.30	1.72					
Christian straight men’s maternal grandmother	871	3.10	1.64	110,350.5	870	.843	.01	.00
Christian straight men’s paternal grandmother		3.10	1.66					
Atheist gay men’s maternal grandmother	239	2.77	1.43	6,339.5	238	.398	−.07	.04
Atheist gay men’s paternal grandmother		2.85	1.40					
Atheist straight men’s maternal grandmother	4,003	2.74	1.42	1,923,840	4,002	.747	.01	.01
Atheist straight men’s paternal grandmother		2.72	1.44					

^a^ This relationship was preregistered for a one-tailed test. *p* value after Benjamini–Hochberg correction also equals .996

### Relationship Between the Degree of Homosexual Attraction and Fertility of Participants and Their Relatives

**Fig. 1:**
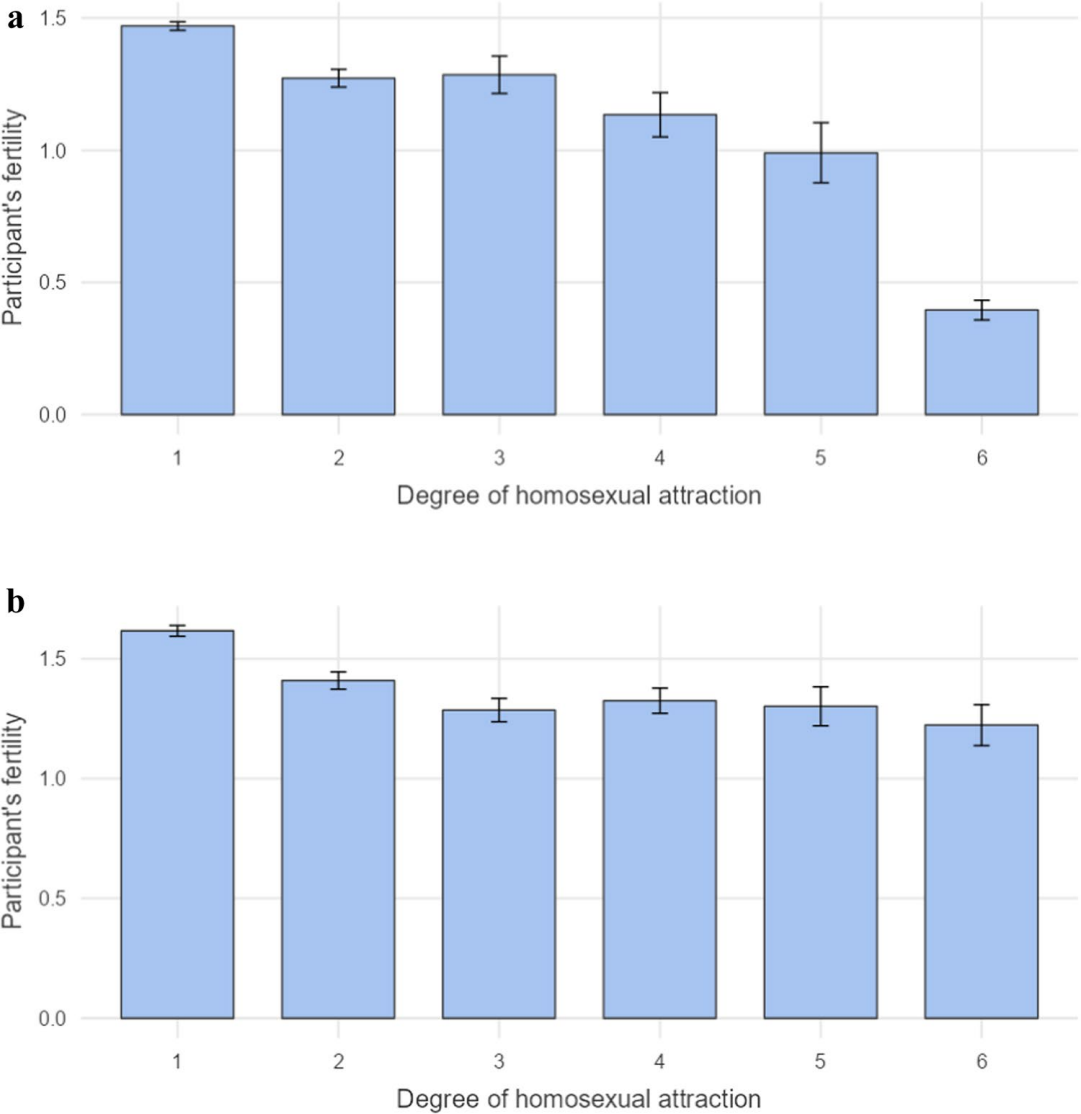
**a** The mean number of children in men with various degrees of homosexual attraction. Error bars represent standard error of the mean. **b** The mean number of children in women with various degrees of homosexual attraction. Error bars represent standard error of the mean

In many analyses, homosexuality is treated as a binary variable. But one can also consider and measure the degree of homosexual attraction on an ordinal scale. We hypothesized that the ordinal measure would deliver more information and tests that use it should be therefore more sensitive than tests that rely on the binary measure. Results concerning the ordinal measure of sexual orientation (degree of homosexual attraction) are summarized in Table 4. For men, we found a significant negative correlation between the degree of homosexual attraction and both the father’s fertility (*τ* = −0.02, *p* = 0.039) and participant’s fertility (*τ* = −0.06, *p* < 0.001), where fertility decreased with an increasing degree of participant’s homosexual attraction. For women, we found significant negative correlations between the degree of homosexual attraction and mother’s fertility (*τ* = −0.02, *p* = 0.025), father’s fertility (*τ* = −0.03, *p* = 0.006), and participant’s fertility (*τ* = −0.03, *p* = 0.008), where the last-named fertility again decreased with an increasing degree of participant’s homosexual attraction. There were no other significant associations. Description of fertility differences in participants according to the degree of their homosexual attraction is depicted in Fig. 1a, b.

**Table 4:** Correlation results utilizing the ordinal definition of sexual orientation

		Fertility				
		Participant	Mother	Father	Maternal grandmother	Paternal grandmother
Men	Partial Kendall *τ*^a^	−.06	−.01	−.02	.00	−.01
	*p*	** < .001**	.063	**.039**	.672	.542
	*N*	6,378	6,908	6,908	6,368	6,319
Women	Partial Kendall *τ*^a^	−.03	−.02	−.03	.00	−.01
	*p*	**.008**	**.025**	**.006**	.705	.464
	*N*	3,172	3,897	3,897	3,735	3,705

Note: All analyses were controlled for participant’s age, size of childhood place of residence, strength of religious belief, and the degree of heterosexual attraction

^a^ A positive correlation denotes a positive relationship between fertility and participant’s homosexual attraction (see Measures in the Method section)

## Discussion

Based on an online collected sample of homosexual and heterosexual men and women, we tested differences in the fertility of participants with various sexual orientations and investigated differences between the fertility of their relatives. We also examined possible differences between the fertility of participants’ maternal and paternal kin. Our results did not support the SAGH: We found no evidence of the fertility of gay men’s mothers and maternal grandmothers being any higher than the fertility of straight men’s mothers and maternal grandmothers. We did, however, observe a slightly higher fertility among gay men’s paternal grandmothers as compared to straight men’s paternal grandmothers, although the effect size was very weak (*τ* = 0.03). Furthermore, we observed lower fertility in mothers and fathers of lesbian women as compared to the parents of straight women. We have also found that homosexual individuals themselves, both gay men and lesbian women, have fewer offspring than heterosexual men and women, whereby the degree of homosexual attraction correlates negatively with fertility in both women and men.

### Lower Fertility of Gay Men and Lesbian Women in Comparison with Their Straight Counterparts

We found that gay men and lesbian women have lower fertility than their straight counterparts; this finding is in line with numerous other studies (Apostolou, 2022; Camperio Ciani et al., 2009, 2018; Coome et al., 2020; Iemmola & Camperio Ciani, 2009; King et al., 2005; Schwartz et al., 2010). We have also found that the difference in fertility of lesbian vs. straight women was smaller (*τ* = −0.03) than the difference in fertility of gay vs. straight men (*τ* = −0.23). This was also reported in Study 1 (but not Study 2) of Apostolou (2022). This association, with a similar magnitude, also held in the subsamples of atheist and Christian men.

### No Differences in the Fertility of Mothers and Maternal Grandmothers between Gay and Straight Men

Our results, which showed that the mothers and maternal grandmothers of gay men were not more fertile than the same classes of relatives of straight men, contrast with the findings of studies that did find support for the SAGH by observing a higher fertility in gay men’s mothers, maternal grandmothers, or both. Such results were reported by Camperio Ciani et al. (2004), Iemmola and Camperio Ciani (2009), Camperio Ciani et al. (2009), Camperio Ciani and Pellizzari (2012), Semenyna et al. (2017), and, for transgender androphilic males, Gómez Jiménez et al. (2020). Other studies, on the other hand, found no support for the SAGH (e.g., Gómez Jiménez et al., 2020 for cisgender androphilic males, Rahman et al., 2008 for white men, Blanchard et al., 2020), or even reported the opposite of what the SAGH predicts, that is, a lower fertility of homosexuals’ female relatives (for instance, Ablaza et al., 2022 or Rahman et al., 2008 for non-white men).

It is possible that contemporary modern societies with low reproduction rates are not good sample populations to test the SAGH and some indigenous, high-fertility populations would deliver different findings—although it should be noted that even in such populations, the results are mixed (Gómez Jiménez et al., 2020; Semenyna et al., 2017). That is why we decided to test our assumptions separately on Christians, where we expected to find a generally higher fertility than in the subsample of atheists. In the Christian subsample, though, the tested relationships opposed the predictions of the SAGH even more: We found that maternal grandmothers of Christian gay men were less fertile than maternal grandmothers of straight Christians. Our assumption that higher fertility in the subsample of Christians and their relatives would be more appropriate for testing the SAGH thus found no support in the empirical data.

### Higher Fertility of Gay Men’s Paternal Grandmothers

In the sample as a whole, we found the paternal grandmothers of gay men to be more fertile than the paternal grandmothers of straight men. This effect was also significant in the subsample of atheists but not in the subsample of Christians. This very weak effect is in line with some other studies (King et al., 2005; Schwartz et al., 2010) and could actually be in line with the SAGH. As Fry (2010) argued, sexually antagonistic selection could in fact work not only for X-based loci but also for autosomal loci. If that is the case, it could be expressed as a higher fertility of gay men’s paternal grandmothers. However, we prefer not to attribute greater significance to such a subtle effect.

The (preregistered) one-tailed test did not find higher fertility in gay men’s maternal grandmothers compared to their paternal grandmothers. It should be noted that had we used a two-tailed test instead, it would have returned a significant result (*p* = 0.007), reflecting a higher fertility of gay men’s paternal grandmothers. A higher fertility of gay men’s paternal grandmothers (as compared to gay men’s maternal grandmothers) was observed in the subsample of Christian gay men. These findings contrast with at least one other study (Camperio Ciani & Pellizzari, 2012) and with what the X-linked version of the SAGH would predict, but they could be explained by sexual antagonism working for autosomal loci (Fry, 2010; see above).

### Correlation Between the Degree of Homosexual Attraction and the Fertility of Participants and Their Relatives

For the female sample, aside from the negative correlation between women’s fertility and the degree of their homosexual attraction, we also found two other significant correlations, namely negative correlations with both the mother’s and father’s fertility and the degree of participant’s homosexual attraction. For the male sample, we found a negative correlation between the father’s fertility and the degree of participant’s homosexual attraction and between the participant’s fertility and the degree of the participant’s homosexual attraction. This effect, however, was weaker than the association between participant’s fertility with binary sexual orientation in the male sample.

### Differences in the Fertility of Relatives between Lesbian and Straight Women

In comparison with straight women’s parents, we observed lower fertility of lesbian women’s mothers and fathers. These results (together with correlations that used the ordinal definition of homosexuality, see above) contrast with those of Camperio Ciani et al. (2018), who found that lesbian women have a significantly larger pedigree size and higher kin fertility. Our results do not support the SAGH or any other balancing mechanism accounting for the reduced reproduction levels of lesbian women. It is possible that female homosexuality was not ancestrally associated with any direct fitness decrease. If this is the case, no evolutionary mechanism to counter the decreased fitness would have needed to emerge. Even in our sample, the fertility of lesbian women was not as decreased as the fertility of gay men (compared to straight individuals of the corresponding sex).

### Summary

Our data did not support the SAGH (at least its commonest, X-linked version) as a solution to the evolutionary conundrum of homosexuality but there are some alternative explanations worth considering. One such is the kin selection model, which posits that homosexual individuals increase inclusive fitness by helping their family members with raising their children (Wilson, 1975, 1978), but studies which tested this hypothesis yielded mixed results (Bobrow & Bailey, 2001; Gómez Jiménez & Vasey, 2022; Vasey et al., 2007).

A related theory proposes that in men, homosexuality could result from unintentional manipulation by some of the homosexual man’s relatives, such as parents or older brothers. This idea is indirectly supported by the finding that gay men have more older brothers than straight men (Blanchard, 2018). The relatives might benefit from enhanced fitness if the sexual orientation of younger brothers is more likely to be shifted toward homosexuality, thereby reducing competition in multi-son families and redirecting parental investment in a way that benefits the fitness of the relevant individuals, i.e., either the parents (Trivers, 1974) or the older brothers (Apostolou, 2013; Flegr, 2022). From the parent’s perspective, having a homosexual son who helps to raise siblings could be advantageous given that parents share a greater portion of alleles with their children (one half) than with their grandchildren (one fourth, Trivers, 1974). For older brothers, the theory is more relevant to originally agricultural societies where inheritance was paramount and brothers competed for a substantial share to increase their attractiveness as potential husbands. If there were too many straight brothers in a family, the probability of each acquiring enough parental wealth decreased. In such environmental conditions, any biological mechanism that increases the probability of homosexuality among later-born male siblings (e.g., increasing exposure to anti-Y antibodies from mothers, see Blanchard & Klassen, 1997 and Bogaert et al., 2018) is more likely to persist despite its reproductive costs. This is because having a later-born homosexual rather than heterosexual male in such contexts might: (1) reduce competition for inheritance among the brothers (see Apostolou, 2013) and potentially (2) increase the survival of the older brothers’ offspring via elevated kin-directed altruism among the younger homosexual brother (see Gómez Jiménez & Vasey, 2022). Altogether, this would increase the direct fitness of the older brothers and the inclusive fitness of the homosexual male. While these hypotheses are intriguing, there is at present insufficient empirical evidence to confirm or refute them.

Another perspective is rooted in the same-sex affiliations hypothesis. This suggests that non-exclusive homosexual behavior, which does not preclude direct reproduction, has certain adaptive benefits. It posits that individuals who engage in homosexual behavior are more inclined to support one another in competition and resource acquisition (Kirkpatrick, 2000; Muscarella, 2000). While supporting evidence in humans remains limited (Fleischman et al., 2015), observations in nonhuman primates provide some validation. For instance, female bonobo dyads which frequently engage in sexual activities are more likely to support each other in intrasexual coalitions (Moscovice et al., 2019). A recent study reinforces this viewpoint, suggesting that in mammals same-sex sexual behavior has evolved to cement social bonds and alliances within groups (Gómez et al., 2023).

We are not aware of any reason why the role of sexually antagonistic selection as a general force promoting *exclusive* homosexuality should be restricted to humans. There is plenty of evidence of non-exclusive homosexual behavior in other animal species (e.g., Gómez et al., 2023; Monk et al., 2019; Vasey, 1995), while exclusive homosexual orientation seems to be almost nonexistent (except for rams, see Roselli, 2020). But why would sexually antagonistic selection promote higher fertility in one sex and sporadic exclusive homosexuality in the other sex only in one of the many possible species? Perhaps the mechanism compensating for the lower reproduction of exclusively homosexual humans depends on another factor that is specific to humankind. Apostolou (2013) proposed that it might hinge upon the human ability to accumulate material possessions and allocate them to one’s heirs, while Barthes et al. (2013, 2015) speculated it may also be related to the social stratification of many human societies.

Another possible explanation for our null results is the hypothesized existence of different biodevelopmental subgroups of male homosexuality (Swift-Gallant et al., 2019; VanderLaan et al., 2022). If this is the case, it would be conceivable that various subgroups of gay men (differing with respect to the biodevelopment of their homosexuality) evolved different ultimate mechanisms to compensate for the lowered reproduction rates (Raymond & Crochet, 2023). This especially intriguing possibility could result in null findings in a study that includes participants with different biodevelopmental trajectories in a single research sample. The SAGH relies on an X-chromosome-related genetic mechanism. If we wanted to restrict our sample only to gay men with a possible genetic etiology of homosexuality (Swift-Gallant et al., 2019), we would have to work with a sample of gay men who have a homosexual relative. Having a homosexual relative is considered one of the biological correlates of homosexuality, or rather a proxy marker for having homosexual alleles. This is also why it could be a good criterion for enriching the study with gay men who have homosexual alleles: They would form a better study sample to test the SAGH. Unfortunately, we did not have information on the sexual orientation of our participants’ relatives. Nevertheless, we suggest that future studies should test the SAGH using a sample of gay men who have at least one homosexual male relative.

Yet another possibility is that the SAGH, as a theoretical construct, is simply not correct, or at least is not demonstrable in modern-day low-fertility populations with their extensive use of contraceptives and family planning, i.e., features which were absent for most of human evolutionary history. We also acknowledge the potential role of gene–environment interactions. Certain genes, while neutral or beneficial for fertility, may interact with specific environmental factors and subsequently influence the likelihood of homosexual orientation and/or fertility, thus adding a layer of complexity to this issue.

We note that our study is one of several recent works that failed to bring evidence in support of the SAGH, that is, the hypothesis of higher fertility of female relatives of homosexual men (Ablaza et al., 2022; Blanchard et al., 2020; Fořt et al., 2024; Raymond et al., 2023; Semenyna et al., 2023; Zietsch et al., 2021). These studies sampled participants from geographically variable populations and used differing methodologies.

Perhaps it is that the number of offspring itself need not be a good indicator of biological fitness in modern times. In this context, the study of Zietsch et al. (2021) is particularly relevant: While they found no genetic correlation between female fertility and the possession of alleles predisposing men to homosexual behavior, they did find such correlation between the number of opposite-sex sexual partners of women and the possession of alleles predisposing men to homosexual behavior (see Introduction). Perhaps the number of opposite-sex sexual partners is thus a better proxy for fitness in societies that use family planning strategies and where the use of contraceptives is widespread. To conduct such study would be rather difficult, but we would like to encourage other researchers to investigate not only the number of offspring but also the number of sexual partners in the relatives (possibly siblings or parents) of gay and straight men and women by inquiring them directly.

### Strengths and Limitations

Our study had several strengths but also some limitations. The main strength is that our questionnaire was completely anonymous and since our participants were not in any way reimbursed for participation, we attracted no semi-professional responders who might be attracted by financial profit. Moreover, our large initial sample size enabled us to narrow down the pool of analyzed individuals to 7,771 men and 3,431 women who were 30 years or older to ensure that participants’ parents and grandparents had already finished their reproduction. Another strength is that we examined fertility associations using both a binary definition of sexual orientation (gay/lesbian and straight individuals) and an ordinal definition of sexual orientation (the degree of homosexual attraction controlled for the degree of heterosexual attraction).

Limitations of our study include the self-selection of participants, i.e., the non-randomness of the sample. Due to the nature of our method, an anonymous internet survey, we were unable to cross-check the self-reported fertility data against reports from other family members. While we recognize the lack of cross-checked self-reports as a shortcoming, this is something that probably applies to most, if not all, studies in this field (e.g., Camperio Ciani et al., 2004, 2009; Gómez Jiménez et al., 2020; Iemmola & Camperio Ciani, 2009; Schwartz et al., 2010; Semenyna et al., 2017).

In our investigation, we employed measures that primarily reflect direct fitness (the counts of offspring and relatives). While this approach is widely used and favored for its clarity and technical feasibility, it need not fully capture the web of relatedness involved in the concept of inclusive fitness. Inclusive fitness becomes especially pertinent when exploring the complexities of gene propagation within pedigrees. Future studies should develop and use a methodology that focuses more on issues of inclusive fitness and relatedness proportions rather than on direct fitness.

It is also possible that our data were enriched with participants from smaller families: It may perhaps be more difficult for individuals from large families to recall all their aunts and uncles (which equals to paternal or maternal grandmother’s fertility), including those they are not in touch with, which is why they left blank the relevant items on the number of their family members. If, for example, some effect connected with sexual antagonism manifested itself only if an individual produced at least a certain liminal number of children, this would hamper our efforts to record such an effect, especially given that we may have selectively lost data on grandmothers’ fertility from participants from larger families. We believe, however, that this limitation is unlikely to have affected the data on mother’s and father’s fertility, because one can quite safely suppose that participants recall the number of their own siblings more easily and accurately than the number of their aunts and uncles.

Another important limitation is that we completely lacked data on the number of participants’ cousins and could not therefore test for differences in the fertility of aunts and uncles of gay vs. straight individuals, as other researchers did (e.g., Camperio Ciani et al., 2004; Gómez Jiménez et al., 2020; Iemmola & Camperio Ciani, 2009). We monitored and controlled for a wider range of potentially confounding variables than is usual in similar studies. Still, some unknown factors may also affect fertility or homosexuality, and this could obscure the relation between our focal variables, increasing the risk of false negative results of statistical tests.

### Conclusions

In a large online collected sample of homosexual and heterosexual men and women, we confirmed that gay men and lesbian women have significantly fewer offspring than their straight counterparts. In line with recent studies, our results do not support the sexually antagonistic gene hypothesis, that is, the notion that maternal female relatives of gay men should be more fertile than the same relatives of straight men. The effect sizes of other observed significant associations in our study were small or very small. It is possible that the number of children is not a good indicator of biological fitness in Western societies and the number of opposite-sex sexual partners should be used instead. The absence of significant effects might be possibly attributed to the hypothesized existence of distinct biodevelopmental subgroups of homosexuality, each with possibly different ultimate explanations.

## Footnotes

1 Throughout the article, we treat the distinctions homosexual vs. heterosexual and gay, lesbian vs. straight as synonymous.

## Declarations

### Data Transparency

Data associated with this research and the preregistration are available publicly at Open Science Framework: https://osf.io/2umzt/
